# Comparing survival outcomes between neoadjuvant and adjuvant chemotherapy within T2N1M0 stage hormone receptor-positive, HER2-negative breast cancer: a retrospective cohort study based on SEER database

**DOI:** 10.1007/s12282-024-01583-5

**Published:** 2024-04-21

**Authors:** Yuhang Shang, Xuelian Wang, Yansong Liu, Weilun Cheng, Yunqiang Duan, Zhengbo Fang, Jiangwei Liu, Fanjing Kong, Ting Wang, Tianshui Yu, Anbang Hu, Jiarui Zhang, Hanyu Zhang, Mingcui Li, Zhiyuan Rong, Yanling Li, Suborna S. Shakila, Xinxin Li, Jianyuan Feng, Fei Ma, Baoliang Guo

**Affiliations:** Department of General Surgery, The Second Affiliated Hospital of Harbin Medical University, Harbin, 150081 China

**Keywords:** Hormone receptor-positive breast cancer, Neoadjuvant chemotherapy, Adjuvant chemotherapy, Survival outcomes, SEER database

## Abstract

**Background:**

Guideline recommendations for the application of neoadjuvant chemotherapy (NACT) in T2N1M0 stage hormone receptor-positive, HER2-negative (HR + /HER2-) breast cancer are ambiguous. The debate continues regarding whether NACT or adjuvant chemotherapy (ACT) offers superior survival outcomes for these patients.

**Materials and Methods:**

Female patients diagnosed with HR + /HER2- breast cancer at T2N1M0 stage between 2010 and 2020, were identified from the Surveillance, Epidemiology, and End Results database and divided into two groups, the NACT group and the ACT group. Propensity score matching (PSM) was utilized to establish balanced cohorts between groups, considering baseline features. Kaplan–Meier (K-M) analysis and the Cox proportional hazards model were executed to assess the efficacy of both NACT and ACT in terms of overall survival (OS) and breast cancer-specific survival (BCSS). A logistic regression model was employed to examine the association between predictive variables and response to NACT.

**Results:**

After PSM, 4,682 patients were finally included. K-M curves showed that patients receiving NACT exhibited significantly worse OS and BCSS when compared with patients undergoing ACT. Multivariable Cox analysis indicated that not achieving pathologic complete response (non-pCR) after NACT (versus ACT), was identified as an adverse prognostic factor for OS (HR 1.58, 95% CI 1.36–1.83) and BCSS (HR 1.70, 95% CI 1.44–2. 02). The logistic regression model revealed that low tumor grade independently predicted non-pCR.

**Conclusion:**

Among T2N1M0 stage HR + /HER2- patients, OS and BCSS of NACT were inferior to ACT. Patients who attained non-pCR after NACT demonstrated significantly worse survival outcomes compared with those who received ACT.

## Introduction

Neoadjuvant or preoperative chemotherapy (NACT), defined as the administration of chemotherapy before surgery, was initially the standard treatment for patients with locally advanced breast cancer. Currently, it is becoming a commonly used option for T2 stage operable (N0-1M0) breast cancer patients [1, 2]. Compared with the traditional strategy of surgery followed by adjuvant chemotherapy (ACT), NACT holds the potential to reduce the tumor size and downstage the nodal involvement, thereby decreasing surgical morbidity and preserving a good appearance [3, 4]. Conversely, NACT might elevate the risk of disease progression and diminish survival time, particularly in chemoresistant tumors, due to the surgical delay [5, 6].

Hormone receptor-positive, HER2-negative (HR + /HER2-) breast cancer, accounting for approximately 70% of all invasive breast cancer cases, is the most prevalent molecular subtype and is also responsible for the majority of breast cancer-related deaths [7, 8]. Studies have reported that HR + /HER2- tumors display less chemosensitivity to NACT compared with other biological subtypes [9, 10]. Current guidelines did not provide conclusive recommendations concerning NACT for T2 stage operable HR + /HER2- breast cancer patients [11–14]. Generally, for patients with T2 stage operable HR + /HER2- breast cancer, primary surgery followed by ACT is preferred over NACT due to the relatively low rate of pathologic complete response (pCR) with NACT [15]. However, in patients characterized by positive lymph nodes (T2N1M0), NACT is still considered for downstaging the tumors and nodes, thus providing more favorable surgical alternatives [12]. Treatment choices primarily rely on patients’ preferences and clinical assessments made by surgeons, while the survival outcomes of T2N1M0 stage HR + /HER2- breast cancer patients undergoing NACT compared with ACT remain uncertain [16].

Previous studies have addressed that NACT was as effective as ACT concerning survival and overall disease progression among all breast cancer subtypes with operable tumors [1, 17, 18]. However, these early studies did not identify breast cancer subtypes. In a recent study, survival outcomes among older women with stage I–II breast cancer who received NACT versus ACT were compared, and no significant differences in survival were observed among HR + /HER2- patients [19]. Nevertheless, the stage I–II cohort was excessively diverse, including both T1 stage tumors that were not generally recommended for preoperative chemotherapy and T3 stage tumors for which NACT was the standard treatment approach [15, 20]. To date, limited data has compared the outcomes of NACT and ACT specifically in T2N1M0 stage HR + /HER2- breast cancer.

Therefore, using the SEER database, we aimed to compare the survival outcomes of HR + /HER2- breast cancer patients at the T2N1M0 stage who underwent NACT with those who received ACT. In addition, we further explored the characteristics of T2N1M0 stage patients who should not routinely adopt NACT.

## Materials and methods

### Data source and study population

**Fig.1:**
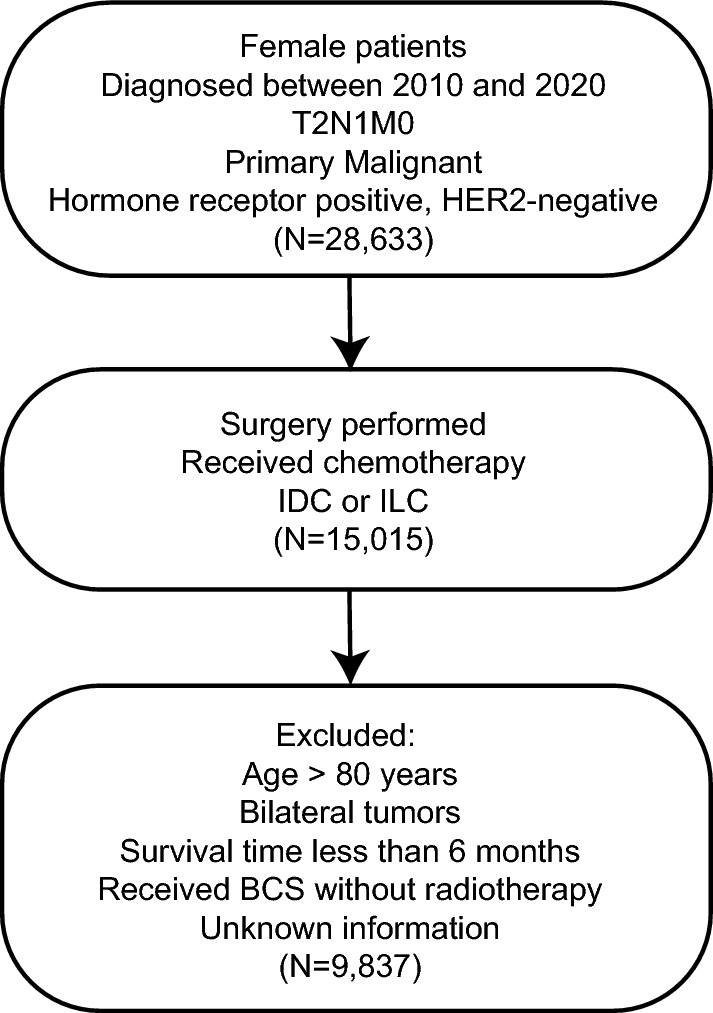
The flowchart of data extraction. *BCS* breast-conserving surgery, *ACT* Adjuvant chemotherapy, *NACT* Neoadjuvant chemotherapy

The Surveillance, Epidemiology, and End Results (SEER) database is currently the largest publicly available cancer database, with information from 18 states that represent all regions of the country [21]. In this retrospective cohort study, we extracted data from 17 registries of the SEER database released in April 2023. 9,837 female HR + /HER2- breast cancer who received surgery and chemotherapy after being diagnosed with primary T2N1M0 invasive ductal carcinoma (IDC) or invasive lobular carcinoma (ILC) between 2010 and 2020 were enrolled. Patients over 80 years old; with bilateral breast cancer; with more than one primary cancer; with unknown information on essential parameters; undergoing breast-conserving surgery (BCS) without radiotherapy; and subject to death or loss to follow-up within 6 months after diagnosis were excluded from the study (Fig. 1).

### Demographic and clinicopathological information

Information on age at diagnosis (15–79 years old), year of diagnosis (2010–2020), marital status (Married, Single, Divorced/separated and Widowed), race (White, Black, Other (American Indian/AK Native, Asian/Pacific Islander)), histology (invasive ductal carcinoma (IDC) and invasive lobular carcinoma (ILC)), grade (I, II, III, and IV), N stage (N1), surgery (breast surgery and axillary surgery), systemic therapy (NACT and ACT), response to neoadjuvant chemotherapy (neoadjuvant chemotherapy not given, Complete Response, Partial Response, No Response and Response but not noted if Complete or Partial) and follow-up were extracted from the SEER database. Following the “Breast Subtype (2010 +)” of the SEER database, HR + was defined as a positive status for estrogen receptor (ER) or (and) progesterone receptor (PR). Pathological ER, PR, and HER2 status were defined according to the American Society of Clinical Oncology and the College of American Pathologists guidelines [22, 23]. Based on the Surgery Codes of the SEER program, we divided the breast surgical procedures into two categories: BCS and Mastectomy. Similarly, the axillary surgical procedures were classified into two categories: no surgery (No) and axillary lymph node dissection (ALND). The age of 50 served as the delineating threshold for classifying menopausal status. Participants were classified as premenopausal if below 50 years and postmenopausal if aged 50 or above [24]. Grades I and II were defined as Low, while grades III and IV were as High. The term "response to neoadjuvant chemotherapy" was the impact of neoadjuvant chemotherapy on the breast. “Neoadjuvant chemotherapy not given” was equal to “adjuvant chemotherapy” in our study. To ensure a conservative estimate, we reclassified the cases labeled as “Response but not noted if Complete or Partial” as “Partial Response”. Then, the records of “Partial response” and “No response” were regarded as not achieving pCR (non-pCR).

### Exposures

The research intervention was NACT, and the control group received ACT. The administration of chemotherapy before surgery was defined as NACT, whereas chemotherapy given after surgery was referred to as ACT.

### Outcomes

We ascertained mortality status by 6 months after diagnosis through December 31, 2020. Overall survival (OS) and breast cancer-specific survival (BCSS) were the outcomes of this study. The causes of death were based on the code of “Vital status recode” and “SEER cause-specific death classification” in the SEER database. OS was defined as the time from the date of diagnosis to the date of death caused by any cause or the most recent follow-up. BCSS was calculated as the time interval between the date of diagnosis and the date of death attributed solely to breast cancer or the most recent follow-up.

### Statistical analysis

All feature variables and clinical parameters in this study were categorical. Baseline comparisons between the NACT and ACT groups were assessed using chi-square tests. A 1:1 nearest-neighbor propensity score matching (PSM) analysis with a caliper of 0.05 was executed through a logistic regression model, incorporating the variables that exhibited significant differences between the two groups in the chi-square tests as covariates. The resulting score-matched cohorts were utilized in subsequent analyses.

Kaplan–Meier (K-M) curves were employed to illustrate the OS and BCSS of study participants, with a log-rank test determining statistical differences between groups. Furthermore, Cox proportional hazard regression models were used to estimate the hazard ratios (HRs) and 95% confidence intervals (CIs) for OS and BCSS. Both univariate and multivariate Logistic regression analyses were conducted to pinpoint factors potentially associated with non-pCR after NACT.

Significance was set as two-sided *p* < 0.05. All analyses were performed using R version 4.3.1.

## Results

### Patients’ characteristics before and after PSM

A total of 9,837 patients were initially identified, including 2346 patients receiving NACT and 7491 patients receiving ACT, and their baseline clinicopathological characteristics are presented in Table 1. Patients with premenopausal status (47.5% versus 36.6%, *p* < 0.001), IDC (94.3% versus 87.2%, *p* < 0.001), high tumor grade (49.9% versus 33.9%, *p* < 0.001), and those who were single (22.4% versus 17.0%, *p* < 0.001) were more inclined to receive NACT. To minimize the significantly different baseline characteristics, a PSM analysis was adopted. 4682 patients were successfully matched and a favorable balance of clinical and pathological features was reached between the NACT and ACT groups (Table 1).

**Table 1:** Baseline characteristics of the study population according to systemic treatment before and after PSM

Variables	Total before PSM (*n* = 9837)			Total after PSM (*n* = 4682)		
	ACT (*n* = 7491)	NACT (*n* = 2346)	*p* value	ACT (*n* = 2341)	NACT (2341)	*p* value
*Menopausal status* (%)			< 0.001			1.000
Premenopausal (< 50 years)	2744 (36.6)	1115 (47.5)		1111 (47.5)	1110 (47.4)	
Postmenopausal (≥ 50 years)	4747 (63.4)	1231 (52.5)		1230 (52.5)	1231 (52.6)	
*Marital status* (%)			< 0.001			0.999
Married	4862 (64.9)	1428 (60.9)		1429 (61.0)	1428 (61.0)	
Single	1274 (17.0)	525 (22.4)		522 (22.3)	521 (22.3)	
Divorced/separated	917 (12.2)	280 (11.9)		279 (11.9)	279 (11.9)	
Widowed	438 (5.8)	113 (4.8)		111 (4.7)	113 (4.8)	
*Race* (%)			< 0.001			1.000
White	5787 (77.3)	1696 (72.3)		1696 (72.4)	1696 (72.4)	
Black	791 (10.6)	326 (13.9)		322 (13.8)	322 (13.8)	
Other	913 (12.2)	324 (13.8)		323 (13.8)	323 (13.8)	
*Histology* (%)			< 0.001			0.899
IDC	6533 (87.2)	2212 (94.3)		2210 (94.4)	2207 (94.3)	
ILC	958 (12.8)	134 (5.7)		131 (5.6)	134 (5.7)	
*Grade* (%)			< 0.001			0.930
Low	4949 (66.1)	1176 (50.1)		1180 (50.4)	1176 (50.2)	
High	2542 (33.9)	1170 (49.9)		1161 (49.6)	1165 (49.8)	
*Axillary surgery* (%)			0.972			0.924
No	5264 (70.3)	1647 (70.2)		1646 (70.3)	1642 (70.1)	
ALND	2227 (29.7)	699 (29.8)		695 (29.7)	699 (29.9)	
*Breast surgery* (%)			0.466			0.832
BCS	2801 (37.4)	857 (36.5)		864 (36.9)	856 (36.6)	
Mastectomy	4690 (62.6)	1489 (63.5)		1477 (63.1)	1485 (63.4)	

*PSM* Propensity score matching, *NACT* Neoadjuvant chemotherapy, *ACT* Adjuvant chemotherapy, *IDC* Invasive ductal carcinoma, *ILC* Invasive lobular carcinoma, *ALND* Axillary lymph node dissection, *BCS* Breast-conserving surgery. *p* value < 0.05 was considered statistically significant

### Survival analysis among patients treated with NACT or ACT and subgroup analysis

**Fig.2:**
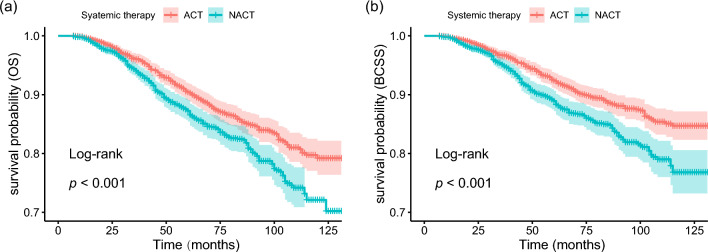
Kaplan–Meier survival curves for OS (**a**) and BCSS (**b**), in patients treated with ACT and those treated with NACT. *ACT* Adjuvant chemotherapy, *NACT* Neoadjuvant chemotherapy, *OS* Overall survival, *BCSS* Breast cancer-specific survival

During a mean follow-up of 131 months, 495 deaths were recorded including 394 deaths from breast cancer and 101 deaths from other causes. K-M curves were employed to access the OS and BCSS of patients. Patients who allocated NACT demonstrated significantly inferior OS (5-year 87.2% versus 90.4%; 10-year 72.1% versus 79.2%, *p* < 0.001) and BCSS (5-year 88.9% versus 92.4%; 10-year 76.8% versus 84.7%, *p* < 0.001) than those who underwent ACT (Fig. 2a–b). Then, we conducted the Cox proportional hazards model for further analysis. Menopausal status, marital status, race, histology, grade, axillary surgery, and systemic therapy were identified as independent predictors of OS. After adjusting for confounding factors, the HR for mortality in the NACT group compared with the ACT group was 1.45 (95%CI 1.26–1.67, *p* < 0.001) (Table 2). In addition, both univariate and multivariate COX regression analysis showed that NACT was an independent prognostic factor of BCSS. Patients who underwent NACT demonstrated a significantly detrimental BCSS (HR 1.56, 95%CI 1.33–1.82, *p* < 0.001) (Table 3). Furthermore, multivariate COX analysis was conducted to examine whether the response to NACT provides additional prognostic information about survival. The findings indicated that no significant difference between the survival outcomes of patients who achieved pCR after NACT and those who received ACT. However, patients who did not achieve pCR had significantly worse OS (HR 1.58, 95%CI 1.36–1.83, *p* < 0.001) and BCSS (HR 1.70, 95%CI (1.44–2.02, *p* < 0.001) than ACT treated patients (Tables 2 and 3).

**Table 2:** Univariate and Multivariate COX regression analysis of OS

Variables	Overall Survival					
	Univariate		Multivariate		Multivariate	
	HR (95% CI)	*p* value	HR (95% CI)	*p* value	HR (95% CI)	*p* value
*Menopausal status* (%)						
Premenopausal (< 50 years)	Ref		Ref		Ref	
Postmenopausal (≥ 50 years)	1.44 (1.26, 1.63)	< 0.001	1.49 (1.31, 1.70)	< 0.001	1.49 (1.30, 1.70)	< 0.001
*Marital status*						
Married	Ref		Ref		Ref	
Single	1.30 (1.11, 1.51)	0.001	1.22 (1.04, 1.43)	0.013	1.21 (1.03, 1.42)	0.017
Divorced/separated	1.23 (1.03, 1.46)	0.022	1.19 (0.99, 1.42)	0.061	1.18 (0.99, 1.41)	0.070
Widowed	1.94 (1.58, 2.39)	< 0.001	1.67 (1.35, 2.06)	< 0.001	1.66 (1.35, 2.06)	< 0.001
*Race*						
White	Ref		Ref		Ref	
Black	1.52 (1.29, 1.78)	< 0.001	1.34 (1.13, 1.58)	0.001	1.34 (1.13, 1.58)	0.001
Other	0.73 (0.59, 0.91)	0.004	0.71 (0.57, 0.88)	0.002	0.71 (0.57, 0.88)	0.002
*Histology*						
IDC	Ref		Ref		Ref	
ILC	0.79 (0.65, 0.96)	0.020	0.94 (0.77, 1.16)	0.581	0.94 (0.77, 1.16)	0.571
*Grade*						
Low	Ref		Ref		Ref	
High	1.74 (1.55, 1.96)	< 0.001	1.70 (1.50, 1.92)	< 0.001	1.73 (1.53, 1.96)	< 0.001
*Axillary surgery*						
No	Ref		Ref		Ref	
ALND	1.28 (1.14, 1.45)	< 0.001	1.27 (1.12, 1.43)	< 0.001	1.26 (1.12, 1.42)	< 0.001
*Breast surgery*						
BCS	Ref					
Mastectomy	1.02 (0.90, 1.15)	0.785				
*Systemic therapy*						
ACT	Ref		Ref			
NACT	1.49 (1.29, 1.71)	< 0.001	1.45 (1.26, 1.67)	< 0.001		
Response to NACT*						
ACT	Ref				Ref	
pCR	1.11 (0.80, 1.55)	0.518			0.98 (0.70, 1.36)	0.894
non-pCR	1.57 (1.36, 1.83)	< 0.001			1.58 (1.36, 1.83)	< 0.001

*NACT* Neoadjuvant chemotherapy, *ACT* Adjuvant chemotherapy, *OS* Overall survival, *HR* Hazard ratio, *CI* Confidence interval, *ref* Reference, *IDC* Invasive ductal carcinoma, *ILC* Invasive lobular carcinoma, *ALND* Axillary lymph node dissection, *BCS* Breast-conserving surgery, *pCR* Pathological complete response, *non-pCR* not achieving Pathologic complete response. *p* value < 0.05 was considered statistically significant. *Systemic therapy, or Response to NACT enrolled in multivariate analysis, not together

**Table 3:** Univariate and Multivariate COX regression analysis of BCSS

Variables	Breast cancer-specific survival					
	Univariate		Multivariate		Multivariate	
	HR (95% CI)	*p* value	HR (95% CI)	*p* value	HR (95% CI)	*p* value
*Menopausal status* (%)						
Premenopausal (< 50 years)	Ref					
Postmenopausal (≥ 50 years)	1.08 (0.94, 1.24)	0.296				
*Marital status*						
Married	Ref		Ref		Ref	
Single	1.31 (1.10, 1.57)	0.002	1.18 (0.99, 1.41)	0.068	1.17 (0.98, 1.40)	0.084
Divorced/separated	1.12 (0.91, 1.39)	0.275	1.12 (0.91, 1.39)	0.283	1.11 (0.90, 1.37)	0.318
Widowed	1.62 (1.25, 2.09)	< 0.001	1.58 (1.22, 2.05)	< 0.001	1.58 (1.22, 2.05)	0.001
*Race*						
White	Ref		Ref		Ref	
Black	1.52 (1.26, 1.84)	< 0.001	1.30 (1.07, 1.58)	0.008	1.30 (1.07, 1.58)	0.007
Other	0.75 (0.59, 0.96)	0.024	0.69 (0.54, 0.89)	0.004	0.69 (0.54, 0.89)	0.004
*Histology*						
IDC	Ref		Ref		Ref	
ILC	0.68 (0.53, 0.86)	0.002	0.92 (0.72, 1.19)	0.542	0.92 (0.72, 1.19)	0.532
*Grade*						
Low	Ref		Ref		Ref	
High	2.15 (1.88, 2.47)	< 0.001	2.04 (1.77, 2.35)	< 0.001	2.08 (1.80, 2.41)	< 0.001
*Axillary surgery*						
No	Ref		Ref		Ref	
ALND	1.28 (1.11, 1.47)	0.001	1.24 (1.08, 1.43)	0.002	1.24 (1.07, 1.42)	0.003
*Breast surgery*						
BCS	Ref					
Mastectomy	1.08 (0.94, 1.25)	0.281				
*Systemic therapy*						
ACT	Ref		Ref			
NACT	1.71 (1.46, 2.00)	< 0.001	1.56 (1.33, 1.82)	< 0.001		
Response to NACT*						
ACT	Ref				Ref	
pCR	1.34 (0.94, 1.91)	0.107			1.03 (0.72, 1.47)	0.877
non-pCR	1.80 (1.52, 2.12)	< 0.001			1.70 (1.44, 2.02)	< 0.001

*NACT* Neoadjuvant chemotherapy, *ACT* Adjuvant chemotherapy, *BCSS* Breast cancer-specific survival, *HR* Hazard ratio, *CI* Confidence interval, *ref* Reference, *IDC* Invasive ductal carcinoma, *ILC* Invasive lobular carcinoma, *ALND* Axillary lymph node dissection, *BCS* Breast-conserving surgery, *pCR* Pathological complete response, *non-pCR* not achieving Pathologic complete response. *p* value < 0.05 was considered statistically significant. *Systemic therapy, or Response to NACT enrolled in multivariate analysis, not together

**Fig.3:**
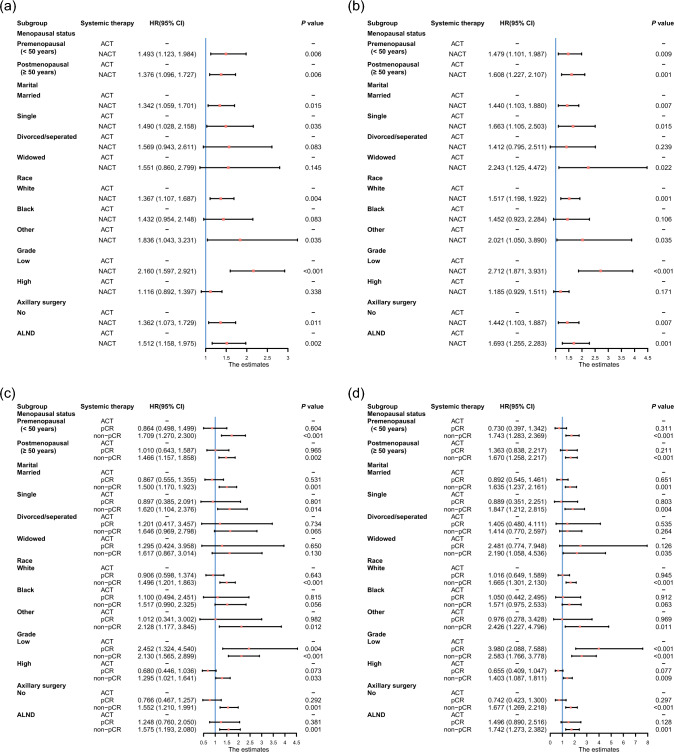
Survival outcome of patients who received ACT and NACT in each subgroup of patients. **a** OS in the Cox proportional hazards model. **b** BCSS in the Cox proportional hazards model. Survival outcome of patients who received ACT and patients who achieved pCR and non-pCR after NACT in each subgroup of patients. **c** OS in the Cox proportional hazards model. **d** BCSS in the Cox proportional hazards model. *ACT* Adjuvant chemotherapy, *NACT* Neoadjuvant chemotherapy, *OS* Overall survival, *BCSS* Breast cancer-specific survival, *pCR* Pathological complete response, *non-pCR* not achieving Pathologic complete response, *HR* Hazard ratio, *CI* Confidence interval

Subsequently, we stratified T2N1M0 stage HR + /HER2- breast cancer patients into subgroups based on significant prognostic factors for survival, including menopausal status, marital status, race, grade, and axillary surgery. Cox analyses were conducted, and forest plots depicting HRs for OS and BCSS were generated to elucidate the prognostic significance of NACT within each subgroup. The results showed a significant difference in HRs for OS and BCSS between NACT and ACT across the majority of subgroups. Notably, patients undergoing NACT displayed higher HRs for both OS and BCSS when compared with those receiving ACT (Fig. 3a–b). Furthermore, non-pCR patients significantly experienced the most unfavorable survival outcomes (Fig. 3c–d).

### Predictive factors of non-pCR

Given the considerable detrimental impact of NACT on survival among non-pCR patients, a logistic regression model was employed to identify the predictive factors for non-pCR in T2N1M0 stage HR + /HER2- breast cancer patients after undergoing NACT. Statistically significant variables (*p* < 0.05) in univariate analysis were enrolled in the multivariate logistic regression model. The outcomes demonstrated that low tumor grade (*p* < 0.001) was an independent predictor of non-pCR (Table 4).

**Table 4:** Univariate and Multivariate logistic regression analysis of factors associated with non-pCR

Variables	Univariate			Multivariate	
	OR(95%CI)	*p* value		OR(95%CI)	*p* value
*Menopausal status* (%)					
Premenopausal (< 50 years)	Ref				
Postmenopausal (≥ 50 years)	1.15 (0.92, 1.42)	0.214			
*Marital status*					
Married	Ref			Ref	
Single	1.21 (0.93, 1.59)	0.171		1.21 (0.92, 1.61)	0.178
Divorced/separated	1.48 (1.03, 2.17)	0.039		1.39 (0.96, 2.07)	0.088
Widowed	1.37 (0.82, 2.45)	0.26		1.23 (0.72, 2.23)	0.471
*Race*					
White	Ref				
Black	0.87 (0.65, 1.19)	0.379			
Others	1.11 (0.81, 1.56)	0.524			
*Histology*					
IDC	Ref			Ref	
ILC	2.64 (1.45, 5.42)	0.004		1.45 (0.77, 3.02)	0.283
*Grade*					
Low	Ref			Ref	
High	0.24 (0.18, 0.30)	< 0.001		0.25 (0.19, 0.32)	< 0.001

*non-pCR* not achieving Pathologic complete response, *OR* Odd ratio, *CI* Confidence interval, *ref* Reference, *IDC* Invasive ductal carcinoma, *ILC* Invasive lobular carcinoma. *p* value < 0.05 was considered statistically significant

## Discussion

In this retrospective study, we evaluated the survival outcomes of individuals diagnosed with T2N1M0 HR + /HER2- breast cancer who underwent either NACT or ACT. Our findings indicated that non-pCR patients who received NACT exhibited inferior OS and BCSS than those who received ACT.

Previous studies have attempted to explore the effectiveness of NACT compared with ACT for early-stage breast cancer. The landmark NSABP B‐18, NSABP B‐27, and EORTC 10902 clinical trials initially revealed that the use of preoperative chemotherapy yielded similar results in terms of progression-free survival and OS compared with conventional postoperative chemotherapy [25–27]. A meta-analysis conducted by the Early Breast Cancer Trialists’ Collaborative Group compared the long-term outcomes of patients with early breast cancer treated with NACT versus ACT. The results demonstrated that no significant difference between NACT and ACT was observed in survival and overall disease progression [28]. Notably, none of the aforementioned studies considered the molecular subtypes of breast cancer or assessed the impact of response to NACT on prognosis.

Herein, we specifically focused on HR + /HER2- patients with T2N1M0 stage tumors to assess the survival outcomes of NACT compared with ACT. Our analysis revealed that ACT was superior to NACT in improving survival outcomes. Findings from stratified analyses across diverse subgroups, including menopausal status, marital status, race, tumor grade, and axillary surgical approach, demonstrated consistency. This could be attributed to the delay in timely surgery. Emerging investigations have revealed that HR + /HER2- tumors exhibit lower sensitivity to NACT compared with other biologic subtypes [29, 30]. Hence, the initial application of NACT is not as effective in tumor eradication as surgery, potentially leading to adverse impacts on survival outcomes [31, 32]. In addition, another factor contributing to reduced OS and BCSS may be the higher likelihood of patients with severe disease receiving NACT. Studies have consistently indicated that patients undergoing NACT present with larger tumors compared with those receiving ACT [10, 19]. Although we attempted to control for factors that could impact survival outcomes in the multivariate model, we did not exclude some unaccounted factors that may potentially influence the selection between NACT and ACT.

pCR demonstrates sensitivity to agents received in the neoadjuvant setting. It has been acknowledged that patients who failed to achieve pCR experienced deleterious long-term outcomes across all breast cancer subtypes [33, 34]. In line with previous research, our study demonstrated that non-pCR after NACT independently predicted an unfavorable prognosis. Patients failing to achieve pCR exhibited the lowest OS and BCSS. Additionally, the pCR rate for the entire cohort was 17.0% (398 out of 2341 patients who received NACT), leaving as much as 83.0% (1943 out of 2341 patients who received NACT) without achieving pCR. Consequently, the high rate of residual disease could be responsible for the compromised survival outcomes among T2N1M0 stage HR + /HER2- breast cancer patients who adopted NACT. The results from the logistic regression model indicated that patients with low tumor grade were more inclined to achieve non-pCR. As a consequence, they should not be routinely recommended for NACT.

Understanding the pathologic response to NACT also provides an opportunity for adjuvant therapy and helps identify high-risk patients for inclusion in novel clinical trials. Patients with residual invasive carcinoma after completing NACT may benefit from subsequent intensive adjuvant therapy. Findings from the CREATE-X and KATERINE clinical trials demonstrated that post-surgical capecitabine and T-DM1, respectively, can improve prognosis in early-stage triple-negative and HER2-positive breast cancer patients when the disease persists after NACT [35, 36]. Furthermore, the MonarchE study revealed that incorporating Aemaciclib into adjuvant endocrine therapy significantly enhanced invasive disease-free survival in patients with HR + /HER2-, node-positive, high-risk early breast cancer, while maintaining an acceptable safety profile. Therefore, adjuvant Aemaciclib may be necessary for patients with T2N1M0 stage HR + /HER2- tumors who did not achieve pCR after NACT. This could represent the potential benefit of NACT for T2 stage operable HR + /HER2- patients.

To our knowledge, this is the first population-based retrospective study examining the prognosis of T2N1M0 stage HR + /HER2- breast cancer patients undergoing NACT or ACT. Leveraging a substantial sample size and extended follow-up duration, our findings carried considerable credibility. Furthermore, we employed PSM to mitigate the impact of confounding variables. Our findings provide valuable insights for guiding the chemotherapy in T2N1M0 stage HR + /HER2- patients. However, the applicability of our findings to populations outside the United States needs to be considered given the variations in demographic traits and healthcare systems. Further multi-center clinical studies are warranted. There are also some limitations in our study. Firstly, despite the ambiguous classification of menstrual status based on RxPONDER clinical trial definitions, the SEER database lacks crucial details essential for HR + breast cancer survival, including reproductive history, family history of breast cancer, endocrine therapy, and specific chemotherapeutic regimens. Additionally, HR + /HER2- breast cancer is predisposed to recurrence, underscoring the importance of studying recurrence risk. Nonetheless, the data lacks information on recurrences and 21-gene recurrence scores (RS) that could predict the magnitude of chemotherapy benefit in early-stage HR + /HER2- breast cancer. Importantly, a high RS can forecast greater chemotherapy benefits, while patients with a low RS receive less benefit without compromising survival [24, 37, 38]. Thus, the absence of this indicator will not influence our primary outcomes. Finally, it is noteworthy that SEER only recorded information about neoadjuvant therapy effects on the primary tumor, while information on the nodal aspect was unavailable. This limitation might affect the assessment of chemotherapy effectiveness and could not be adjusted in the analysis.

## Conclusion

The application of NACT in T2N1M0 stage HR + /HER2- breast cancer patients continues to be a matter of debate. Utilizing the SEER database, our study uncovered that NACT exerted a detrimental impact on survival in comparison with ACT. non-pCR after NACT was a significant prognostic factor for unfavorable survival outcomes. Therefore, the adoption of NACT for T2N1M0 stage HR + /HER2- breast cancer patients warrants careful consideration, particularly for those with low tumor grade, who are more inclined to be non-pCR after undergoing NACT. A prospective randomized controlled trial will be necessary to validate these findings and establish an improved treatment stratification scheme for T2N1M0 stage HR + /HER2- breast cancer patients.

## Data Availability

The datasets analyzed during the current study are available in the SEER database. https://seer.cancer.gov/.

## Abbreviations

NACT: Neoadjuvant chemotherapy
ACT: Adjuvant chemotherapy
HR + /HER2-: Hormone receptor-positive HER2 negative
SEER: Surveillance epidemiology, and end results
ER: Estrogen receptor
PR: Progesterone receptor
OS: Overall survival
BCSS: Breast cancer-specific survival
pCR: Pathologic complete response
non-pCR: Not achieving Pathologic complete response
PSM: Propensity score matching
BCS: Breast-conserving surgery
HR: Hazard ratios
CI: Confidence interval
ALND: Axillary lymph node dissection
IDC: Invasive ductal carcinoma
ILC: Invasive lobular carcinoma
RS: Recurrence scores
